# Review of “*Parasitology: a conceptual approach*” by Eric S. Loker and Bruce V. Hofkin

**DOI:** 10.1186/s13071-015-0905-3

**Published:** 2015-06-10

**Authors:** Filipe Dantas-Torres

**Affiliations:** Department of Immunology, Aggeu Magalhães Research Centre, Oswaldo Cruz Foundation, 50740465 Recife, Brazil; Department of Veterinary Medicine, University of Bari, 70010 Valenzano, Italy

## Abstract

**Loker ES, Hofkin BV:***Parasitology: A Conceptual Approach.* Garland Science, Taylor & Francis Group; 2015. 560 pages. ISBN 978-0-8153-4473-5

## Review

*Homo sapiens* is the most dominant species on earth, isn’t it? If true, why are we still succumbing from parasitic diseases? Why are these ‘unintelligent’ forms of life called parasites still killing thousands of people, despite tens of billion of dollars being poured annually into R & D? Sometimes I think to myself: “parasites are very smart creatures”. And I really believe they are!

I love everything about parasites. They constitute a heterogeneous group of organisms, including protozoans, roundworms, tapeworms, flukes, and arthropods. Each parasite has developed its own set of strategies to find its host (or hosts) and to benefit from it. These strategies results from millions of years of evolution aimed at surviving in a changing world, where only the strongest and the fittest will survive. The book *Parasitology: A conceptual approach*, by Eric S. Loker and Bruce V. Hofkin, addresses this issue.

The book is divided into 10 chapters, each of which subdivided into three to nine subsections. Throughout the first three chapters, the authors provide definitions of traditional and emerging concepts in parasitology (e.g., parasitism, mutualism, commensalism, vector competence, vector capacity, sexual and asexual reproduction, horizontal gene transfer, biological and evolutionary species concepts, and cryptic species). Other relevant issues such as DNA barcoding, metagenomics, intraspecific variation, homoplasy, and monophyly are also touched upon. Types of parasites (endoparasites, ectoparasites, castrators, body snatchers, obligatory, facultative, opportunistic, hyperparasites, macroparasites, microparasites, and protelean parasites), hosts (definitive, intermediate, paratenic and reservoir hosts) and vectors (mechanical and biological) are described. Different parasites within the Eukaryota, are discussed. Besides traditional parasitic groups, the authors included parasitic algae, plants, and fungi. Even bacteria are defined as parasites! The authors also ask thought-provoking questions such as “Do parasites give rise to free-living organisms?” that made my head spin! Such questions may cause vertigo in parasitologists who defend that parasitism is a one-way trip, but may stimulate further research on different models and/or parasitic groups to improve our understanding of the evolutionary biology of parasitism. Indeed, I found the second chapter exciting, but it may be challenging for beginners.

The third chapter is a more traditional one and describes “the parasite’s way of life”. After providing a historical account of pioneer studies on parasite transmission, an overview of selected parasite life cycles is provided. The selected examples are always pertinent, but as a veterinary parasitologist I missed some classical cases such as *Trichinella spiralis*, whose life cycle is sometimes referred to as *auto-heteroxenous* (a concept not approached in the book). Emerging issues such as co-infection, the relationship between genome size and parasitism, epigenetic phenomena and co-opting of host signalling molecules are also addressed.

Chapters 4 to 9 are dedicated to a range of issues, including immunology (host defence and parasite evasion), pathology (and disease), ecology (parasite habitats, population biology, role of parasites in food webs and ecosystems, and parasite diversity), evolutionary biology of parasitism (co-evolution of parasite-host interactions, evolution of virulence, effects of parasites on host evolution), and parasite control (strategies to reduce transmission, anti-parasitic drugs, and vaccines). The tenth chapter brings up questions about the future of our planet if current trends in human development and population growth continue, highlighting research needs, to better understand the ecological and evolutionary roles of parasites as well as to develop improved methods to detect and to control them.

All chapters are rich in figures, tables and boxes. The reading is enjoyable and quality of figures is adequate, which denotes the care of the authors and the professionalism of the publisher. A plus of the book is definitely the *Rogues’ Gallery of Parasites*, which is a more schematic chapter about distribution, prevalence, transmission, pathology, diagnosis, treatment, and control of selected parasites. There are some minor omissions (e.g., species of the genus *Phlebotomus* also transmit *Leishmania* parasites in Europe, and not only in Africa and Asia as stated on page 436) and debatable information (e.g., the inclusion of *Babesia microti* in the genus *Theileira* is still a subject of debate) in this chapter. Nonetheless, the *Rogues’ Gallery of Parasites* provides a well-balanced summary of the selected parasites.

Another interesting feature of the book is the availability of learning and teaching resources in the publisher’s website. Students may find, for example, a tutorial on constructing and using evolutionary trees, a guide to answering the end-of-chapter questions, and a searchable glossary. Instructors may find images from the book and a guide on how to structure a course using the book.

I shall recommend this book for anyone dealing with any aspect of parasitology. It is a rich source of updated information on emerging issues in parasitology, including parasite-host interactions, parasite ecology and evolution. I will certainly put my hardcopy in my bookshelf “must have books on parasitology”.

